# Estimating the Contribution of Proteasomal Spliced Peptides to the HLA-I Ligandome*[Fn FN1]

**DOI:** 10.1074/mcp.RA118.000877

**Published:** 2018-09-05

**Authors:** Roman Mylonas, Ilan Beer, Christian Iseli, Chloe Chong, Hui-Song Pak, David Gfeller, George Coukos, Ioannis Xenarios, Markus Müller, Michal Bassani-Sternberg

**Affiliations:** From the ‡Vital-IT,; §Swiss Institute of Bioinformatics, 1015 Lausanne, Switzerland;; ¶Adicet Bio Israel, Ltd., Technion City, 32000, Haifa, Israel;; ‖Ludwig Cancer Research Center, University of Lausanne, 1066 Epalinges, Switzerland;; **Department of Oncology, University Hospital of Lausanne, 1011 Lausanne, Switzerland

**Keywords:** De novo sequencing, Peptidomics, Bioinformatics searching, Mass Spectrometry, Immunology, Human Leukocyte Antigen, Immunopeptidomics, Proteasome-spliced peptides

## Abstract

Spliced peptides are short protein fragments spliced together in the proteasome by peptide bond formation. True estimation of the contribution of proteasome-spliced peptides (PSPs) to the global human leukocyte antigen (HLA) ligandome is critical. A recent study suggested that PSPs contribute up to 30% of the HLA ligandome. We performed a thorough reanalysis of the reported results using multiple computational tools and various validation steps and concluded that only a fraction of the proposed PSPs passes the quality filters. To better estimate the actual number of PSPs, we present an alternative workflow. We performed *de novo* sequencing of the HLA-peptide spectra and discarded all *de novo* sequences found in the UniProt database. We checked whether the remaining *de novo* sequences could match spliced peptides from human proteins. The spliced sequences were appended to the UniProt fasta file, which was searched by two search tools at a false discovery rate (FDR) of 1%. We find that 2–6% of the HLA ligandome could be explained as spliced protein fragments. The majority of these potential PSPs have good peptide-spectrum match properties and are predicted to bind the respective HLA molecules. However, it remains to be shown how many of these potential PSPs actually originate from proteasomal splicing events.

## Abstract (precis)

It has been reported that about 30% of the HLA-I ligands are produced by proteasomal splicing of two noncontiguous fragments of a parental protein. We report that the identification of many of those spliced peptides is ambiguous. With an alternative workflow, based on *de novo* sequencing and subsequent verification with multiple search tools, we estimate that the upper bound for the proportion of cis-spliced peptides is 2–6%. Nevertheless, the true contribution of spliced peptides to the ligandome may be much smaller.

## Graphical Abstract

**Figure:**
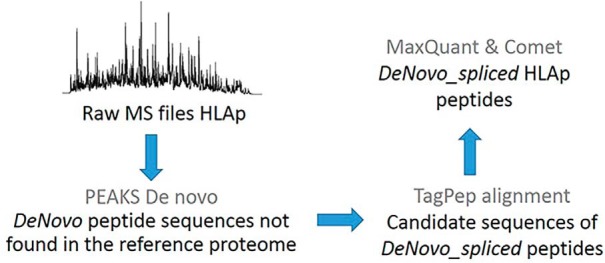

**Highlights**

- Reported proteasomal spliced HLA peptides do not fit the consensus binding motifs.
- Their MS/MS spectrum matches suggest that many of them are ambiguous.
- Our workflow is based on *de novo* sequencing, alignment, and multiple search tools.
- The upper bound proportion of cis-spliced peptides is 2–6% and likely much smaller.

The antigen processing and presentation machinery is responsible for the cell surface display of thousands of peptides in the context of the HLA^1^ class I (HLA-I) molecules. The proteasome is considered as the main protease that cleaves endogenous proteins. However, in addition to the proteasome, the antigen processing and presentation machinery comprises several other proteases, transporters, and chaperones that cooperatively digest the proteins in the cytoplasm, funnel the peptides into the endoplasmic reticulum (ER), further trim and edit them, load them on newly synthesized HLA-I, and finally direct the stable complexes to the cells' surface (1). The selective interaction between the HLA-I complex and the peptides is the major factor that defines the presented repertoire and is often represented with binding motifs.

Currently, the only unbiased methodology to comprehensively interrogate the repertoire of the HLA-I binding peptides (HLA-Ip) is based on mass spectrometry (MS). HLA complexes are immunoaffinity-purified from cells in culture or from tissues; the peptides are extracted and subjected to reverse-phase liquid chromatography (LC) coupled online to sensitive MS instruments. The acquired tandem mass spectrometry (MS/MS) data are normally searched against a database of protein sequences. Applying a stringent FDR of 1% using a comparable decoy database leads to the accurate identification of thousands of HLA-Ip. HLA-Ip are mainly 9–11 amino acids (AA) long, and usually about 95% of the peptides identified with this methodology fit the consensus binding motifs of the HLA-I molecules expressed in the samples (2).

In a recent MS-based HLA-I ligandomics study, a novel computational algorithm predicted that a surprisingly large fraction, up to 30%, of the ligands may be derived from transpeptidation of two noncontiguous fragments of a parental protein that are spliced together within the proteasome (3). Earlier work showed several cases of such proteasomal spliced HLA-I peptides that were naturally presented and recognized by cytotoxic T cells (4–9). Hence, these may be highly interesting therapeutic targets. However, the authors of (3) noticed that unlike the nonspliced peptides, proteasome-spliced peptides (PSPs) had low HLA binding affinities and produced ambiguous binding motifs compared with normal HLA-Ip. HLA loading takes place after the peptides have exited the proteasome and entered the ER and hence lost the identity of their creation mechanism. Currently, there is no mechanism or biological process that could explain how the antigen processing and presentation machinery can prioritize loading of HLA-I molecules with low-affinity PSPs over high-affinity nonspliced peptides.

Understanding the contribution of PSPs to the HLA ligandome is crucial, especially as they may indeed be highly interesting therapeutic targets in many diseases. Here we critically investigated PSPs reported in Liepe *et al.* (3) and found that most of the spectra attributed to them could be assigned with higher scores to normal peptide sequences within the reviewed part of UniProt database (with no isoforms) of the human proteome. We further describe an alternative computational pipeline to estimate the contribution of spliced peptides to the immunopeptidome. Our results suggest that less than 2–6% of the HLA-Ip may be spliced. As opposed to the spliced peptides reported in (3), these peptides fit well to the relevant HLA binding motifs.

## EXPERIMENTAL PROCEDURES

### HLA Ligandomic Data

We selected previously published MS HLA-Ip datasets of exceptionally high coverage representing a variety of binding specificities (supplemental Table 1). MS raw files of HLA-Ip isolated from two melanoma tissues, Mel15 (16 raw files) and Mel16 (12 raw files) (10), RA957 B cell line (four raw files) (11), and fibroblast (Fib) cells (four raw files) (2) were downloaded from the PRoteomics IDEntifications (PRIDE) repository (12) dataset PXD004894, PXD005231 and PXD000394, respectively. One of the four raw MS files of the Fib cells (20130504_EXQ3_MiBa_SA_Fib-2.raw) was also used by Liepe *et al.* More details about these datasets can be found on PRIDE and the respective manuscripts.

### Data Processing

If not otherwise mentioned, data were processed with the R statistical scripting language (version 3.3.2) (https://www.r-project.org/).

### Experimental Design and Statistical Rationale

#### 

##### Identification of HLA-Ip Using PEAKS

Raw files were analyzed with the *de novo* sequencing software PEAKS Studio 8.0 (13). General parameters were set to “Ion Source”: electrospray ionization (ESI; nanospay), “Fragmentation Mode”: high energy Collision-induced dissociation (CID) (y and b ions), “MS Scan Mode,” and “MS/MS Scan Mode”: Fourier-transform ion cyclotron resonance (FT-ICR)/Orbitrap. The different PEAKS modules were used in the following order with their default parameters while special parameters are indicated in parenthesis: 1) DATA REFINE; 2) PEAKS DENOVO (“Parent Mass Error Tolerance”: 10 ppm, “Fragment Mass Error Tolerance”: 0.02 Da, “Enzyme”: None); and 3) PEAKS DB (“Parent Mass Error Tolerance”: 10 ppm, “Fragment Mass Error Tolerance”: 0.02 Da, “Variable Modifications”: Oxidation (M) 15.99, “Database”: Homo_sapiens_UP000005640_9606).

A table containing the five best scoring *de novo* sequences for every spectrum, named “all *de novo* candidates” was exported from PEAKS DENOVO. A table containing peptides with a match to human proteins from UniProt (Homo_sapiens_UP000005640_9606), named “DB search psm,” was exported from PEAKS DB. For this export, all peptides with -10LogP > 15 (FDR around 1.5%) were considered having a match. Subsequent filtering and annotation of the “all *de novo* candidates” table was done using the R statistical scripting language (version 3.3.2).

All peptides in “all *de novo* candidates” with a corresponding match in “DB search psm” were annotated using a new column “database peptide.” If a spectrum had a matching database peptide, then all other peptides corresponding to this spectrum were removed from “all *de novo* candidates”. An additional column “*de novo* only” was added and unmatched sequences were marked with a “+.” Only peptides with a length between 8 and 25 AA and a minimum local confidence score over 80 for every AA position were kept. Peptides containing post-translational modifications (PTMs) were removed in order to simplify association of peptides with their corresponding HLA alleles (supplemental Data 1–4). For the subsequent analysis we merely kept peptides marked as “*de novo* only” and named them “*de novo* only peptides,” and the final table is made available in the supplemental material (supplemental Data 5).

##### Identification of Possible Spliced Peptides Using TagPep

We checked whether the filtered list of sequences from the “*de novo* only peptides”, which did not match any UniProt sequence, could be spliced fragments from UniProt (Homo_sapiens_UP000005640_9606, the reviewed part of UniProt, with no isoforms, including 21,026 entries downloaded in March 2017) proteins. We applied an in-house software tool TagPep, which uses the index strategy described for fetchGWI (14) adapted for AAs instead of nucleotides. TagPep first matches the whole peptide sequence to the database. If there is no complete hit, it looks for hits allowing for one splicing event, where both spliced fragments are from the same protein (supplemental Data 1). We excluded *trans*-spliced peptides where the fragments stem from two different proteins for three reasons. First, all spliced peptides reported in (15) are concatenated fragments from the same protein. Second, the huge number of *trans*-spliced may lead to strongly increased false positive rates in subsequent bioinformatics analysis, and third, for *trans*-splicing to happen, the two source proteins need to be present in the same proteasome at the same time, which is unlikely to happen on a large scale (16). The spliced fragments can lie anywhere in the protein, but their sequences cannot overlap. Within a protein, TagPep prioritizes the spliced peptide with the smallest splicing gap and lists all possible splicing events matching different proteins. PEAKS *de novo* assigns the mass 113.08406 by default as leucine; however, TagPep alignment considered either leucine or isoleucine for possible matches. We named the resulting set of TagPep matched peptides as *DeNovo_spliced*, and these are the subset of potentially proteasome-spliced sequences. For each sample, we provide the PSMs from PEAKS, including the UniProt hits and the “*de novo* only peptides”, and we flagged the potential *DeNovo_spliced* peptides (supplemental Data 5).

##### Confirmation of Identification of Spliced Peptides Using MaxQuant and Comet

We employed the MaxQuant platform (17) version 1.5.5.1 and the Comet software release 2016.01 (18) to search the peak lists against a fasta file containing the UniProt database (Homo_sapiens_UP000005640_9606, the reviewed part of UniProt, with no isoforms, including 21,026 entries downloaded in March 2017) and a list of 247 frequently observed contaminants. For each sample, we added to the fasta file the list of *DeNovo_spliced* peptide sequence candidates. For Fib, we also added the 1,154 spliced peptides identified by Liepe *et al.* (3), which we named *LM_spliced*. The list of spliced peptides was kindly provided to us by the authors of (3) (supplemental Data 6, type = “psp”). Peptides with a length between 8 and 25 AA were allowed. MaxQuant parameters: The second peptide identification option in Andromeda was enabled. The enzyme specificity was set as unspecific. An FDR of 1% was required for peptides and no protein FDR was set. The initial allowed mass deviation of the precursor ion was set to 6 ppm, and the maximum fragment mass deviation was set to 20 ppm. For Fib, methionine oxidation (15.994915 Da) was set as variable modification; however, modified peptides were removed at first for the direct comparison to the Liepe's data. For the additional modification searches in all samples, methionine oxidation, N-terminal acetylation and glutamine/asparagine deamidation (+0.98402 Da) were set as variable modifications. Comet parameters: activation method: HCD, peptide mass tolerance: 0.02 Da, fragment mass tolerance: 0.02, fragments: b- and y-ions, precursor tolerance, and modifications were the same as in MaxQuant settings. The output files summarizing MaxQuant and Comet result files are provided as supplemental Data 7–16, and explanation of the column headers are provided in supplemental Table 2.

##### LC-MS/MS Analyses and Identification of Selected Synthetic HLA-Ip

Synthetic peptides (PEPotech Heavy grade 3, Thermo Fisher Scientific) (listed in supplemental Table 3) corresponding to peptides identified from Fib data were mixed and desalted on a C-18 spin column (Harvard Apparatus, 74–4101) and measured at a total amount of 10 and 20 pmol. Synthetic peptides were separated by a nanoflow HPLC (Proxeon Biosystems, Thermo Fisher Scientific, Odense) and coupled on-line to a Q Exactive HF mass spectrometer (Thermo Fisher Scientific, Bremen) with a nanoelectrospray ion source (Proxeon Biosystems). We packed a 20 cm long, 75 μm inner diameter column with ReproSil-Pur C18-AQ 1.9 μm resin (Dr. Maisch GmbH, Ammerbuch-Entringen, Germany) in buffer A (0.5% acetic acid). Peptides were eluted with a linear gradient of 2–30% buffer B (80% acetonitrile and 0.5% acetic acid) at a flow rate of 250 nl/min over 90 min. Data were acquired using a data-dependent 'top 10' method. We acquired full scan MS spectra at a resolution of 70,000 at 200 *m/z* with an auto gain control target value of 3e6 ions. The ten most abundant ions were sequentially isolated, activated by higher-energy collisional dissociation and accumulated to an auto gain control target value of 1e5 with a maximum injection time of 120 ms. In case of unassigned precursor ion charge states, or charge states of four and above, no fragmentation was performed. The peptide match option was disabled. MS/MS resolution was set to 17,500 at 200 *m/z*. Selected ions form fragmentation were dynamically excluded from further selection for 20 s. We employed the MaxQuant settings mentioned above for synthetic peptides identification. The raw files and MaxQuant output tables have been deposited to the ProteomeXchange Consortium via the PRIDE partner repository with the dataset identifier PXD010793.

##### Comparison of MS/MS Annotations of Endogenous HLA-Ip and Their Synthetic Counterparts

To investigate if spliced peptides match the MS/MS spectra better than possible alternative sequences we first compared the MS/MS scans identified by Liepe *et al.* as spliced peptides and those identified by MaxQuant. The mapping to the relevant MS scans and their Mascot ion scores were kindly provided to us by the authors of (3) (supplemental Data 6). For the Fib data, we selected three MS/MS scans of *LM_spliced* peptides identified both by MaxQuant and by Liepe *et al.*, and 21 MS/MS scans corresponding to *LM_spliced* peptides that MaxQuant identified instead as UniProt peptides. This selection was not biased and was not based on prior knowledge, which would favor MaxQuant. We synthesized the 21 pairs of peptide sequences and the three *LM_spliced* peptide sequences and analyzed them by MS as mentioned above. For visual inspection, we printed the endogenous and synthetic spectra to pdf files. For each of the 21 pairs, we calculated the similarity between the spectrum of the eluted peptide from Fib, annotated as *LM_spliced* and the spectrum of the synthetic *LM_spliced* peptide. We also calculated the similarity between the spectrum of the eluted peptide from Fib annotated as a UniProt peptide, and the spectrum of the synthetic UniProt peptide. Similarly, we calculated the similarity between the three spectra of the identically identified spliced sequences and the spectra of their synthetic counterparts. The similarity was computed by the cosine score (value between 0 and 1, where a value of 0 corresponds to spectra with no peaks in common and a value of 1 to identical spectra) (19). The MzJava class library (20) was used to read the .mgf spectrum files and to calculate the similarity.

##### Binding Affinity Prediction and Clustering of Peptides

We used the NetMHCpan (21) to predict binding affinity of 8–14-mer peptides to the respective HLA alleles expressed in the sample and assigned them based on maximum affinity. Hits with a rank <2% were considered as binders. Gibbscluster-1.1 (22) was run independently for each list of peptides identified from the different samples, with the default settings except that the number of clusters was tested between 1 and 6, a trash cluster was enabled and alignment was disabled (23). The MixMHCp tool (http://mixmhcp.org/) was used to cluster the peptides with default settings (11, 24).

## RESULTS

### 

#### 

##### Predicted Spliced Peptides from Liepe et al. Do Not Fit Well to The Consensus Binding Motifs

**Fig. 1.**
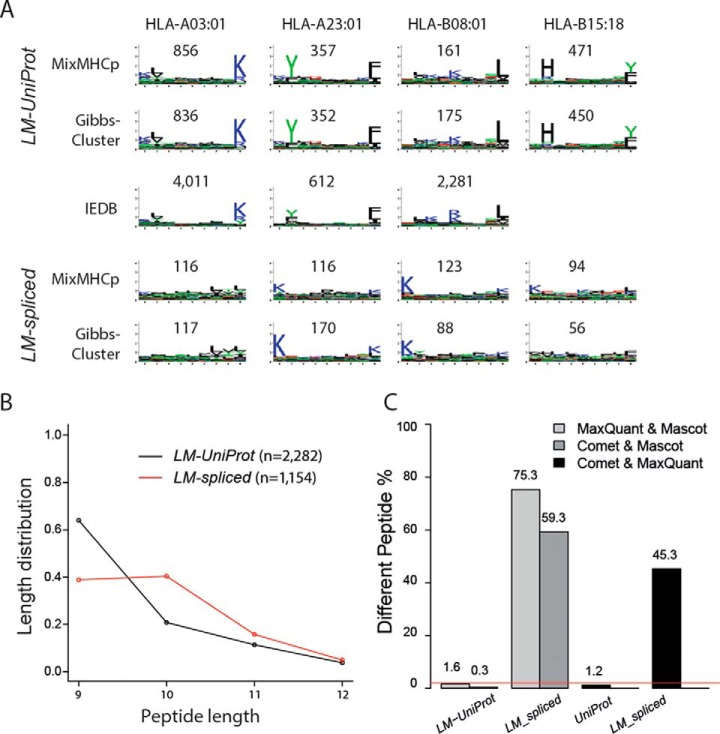
(*A*) Motif deconvolution analysis with MixMHCp and GibbsCluster of the 9-mer *LM_UniProt* and *LM_spliced* peptides, and comparison to known logos from Immune Epitope Database and Analysis resource (IEDB) for HLA-A03:01, HLA-A23:01, and HLA-B08:01 (HLA-B15:18 has no experimental ligands in IEDB). Motifs found in *LM_UniProt* peptides are highly reproducible and comparable to the known motifs from IEDB, while this is not the case for motifs found in *LM_spliced* peptides. (*B*) Length distribution of the *LM_UniProt* and *LM_spliced* peptide. (*C*) Rate of differing PSMs for the peptides in *LM_UniProt* and *LM_spliced*. Only MS/MS scans where both search strategies reported a match at FDR of 1% were considered.

Spliced HLA-Ip identified by Liepe *et al.* in the Fib sample (*LM_spliced*) were reported to be barely compatible with the corresponding HLA-I binding motifs (3). First, we tested to what extent the reported 1,154 *LM_spliced* peptides follow the same binding specificity as the other 2,882 reported HLA-Ip, which matched proteins in UniProt with the Mascot tool in Liepe *et al.* (*LM_UniProt*, type = “pcp” in Supplemental Data). 90% of the *LM_UniProt* peptides were predicted binders by NetMHCpan compared with only 33% of the *LM_spliced* peptides (supplemental Fig. 1*A* and 1*B*). Second, we used the computational tools MixMHCp (11, 24) and GibbsCluster (22) to identify the consensus binding motifs within the lists of 9-mer HLA-Ip in a fully unsupervised way (*i.e.* without predicting their binding affinity). Four dominant motifs corresponding to the HLA-A and HLA-B allotypes expressed in the Fib cells were identified for the *LM_UniProt* peptides with both methods, whereas the motifs found in the *LM_spliced* peptides were much less specific and did not match the known alleles (Fig. 1*A*). This is unlikely related to the relatively lower number of clustered *LM_spliced* peptides, as clustering of stringently identified ligandomic datasets comprising hundreds of peptides is sufficient in most cases to reveal the specific anchor residues (as shown below). Furthermore, we observed a different length distribution of the *LM_spliced* and *LM_UniProt* peptides (Fig. 1*B*). *LM_UniProt* peptides followed the expected peptide length distributions of HLA-I alleles with the majority of peptides of length 9, while *LM_spliced* peptides displayed the same amount of 9- and 10-mers. We obtain similar results for the fraction of 1,583 spliced and 3,779 UniProt 9-mer peptides of the GR-LCL 2D data reported by Liepe *et al.* (supplemental Fig. 1*C* and 1*D*). Altogether, our results show that expected HLA-Ip characteristics can be clearly recovered from the list of UniProt peptides but cannot be observed in the spliced peptides reported by Liepe *et al.*

##### Spliced Peptides from Liepe et al. Produce More Ambiguous PSMs

We checked whether the spliced PSMs reported by Liepe *et al.* could be explained as matches to UniProt peptides. We added the list of 1,154 *LM_spliced* peptide sequences to the reviewed part of the UniProt database (with no isoforms) and re-searched the MS/MS data of the Fib raw file used by Liepe *et al.* using MaxQuant and Comet firstly without considering variable modifications. Out of the 6,033 (7,221) MS/MS scans identified by MaxQuant (Comet) at an FDR of 1%, 3,211 (3,347) MS/MS scans were also matched formerly with the Mascot tool by Liepe *et al.* (Table I). Regarding these common MS/MS, we found very good agreement between MaxQuant and Comet in terms of matches of MS/MS scans to *LM_UniProt*. Only 1.6% (0.3%) of these scans matched a different peptide sequence in the MaxQuant (Comet) search (Fig. 1*C*). Since all searches were performed at a spectrum level FDR of 1%, the differences should not be larger than 2% and these values are within this range. However, for the group of common MS/MS scans, which matched to *LM_spliced* peptides in (3), the disagreement was 75.3% and 59.3% for MaxQuant and Comet, respectively. MaxQuant or Comet matched a UniProt peptide for the majority of these conflicts (91.6% for MaxQuant and 88.0% for Comet) and only in a few cases a different spliced peptide sequence. Furthermore, in the Fib dataset, when we clustered 255 sequences matching *UniProt* proteins by MaxQuant that the same MS/MS scans have been originally identified as *LM_spliced* by Liepe *et al.* (9-mers only), we revealed the expected binding motifs (supplemental Fig. 2*A*). However, when we clustered the 144 *LM_spliced* sequences that MaxQuant matched their MS/MS scans to UniProt peptides (9-mers only), as well as the 449 remaining *LM_spliced* peptides after removal of conflicting PSMs that have been assigned as UniProt by MaxQuant (9-mers only), we again could not reveal the binding motifs (supplemental Fig. 2*B* and 2*C*). Overall, in our reanalysis of the Liepe *et al.* data, 12 and 14% of the *LM-spliced* peptides scored higher than the competing UniProt peptides with MaxQuant and Comet, respectively. More details about these comparisons can be found in Table I. Interestingly, if we compare results between MaxQuant and Comet, the group of MS/MS scans matched to a peptide in *LM_spliced* also show a highly inflated disagreement (45.3%) compared with MS/MS scans matched to UniProt (1.2%) (Fig. 1*C*). Therefore, these results do not depend on the particular choice of search tool, but it seems that the PSMs assigned as *LM_spliced* peptides were more ambiguous compared with UniProt peptides, possibly because many *LM_spliced* peptides bear strong similarity to UniProt peptides.

**TABLE 1:**
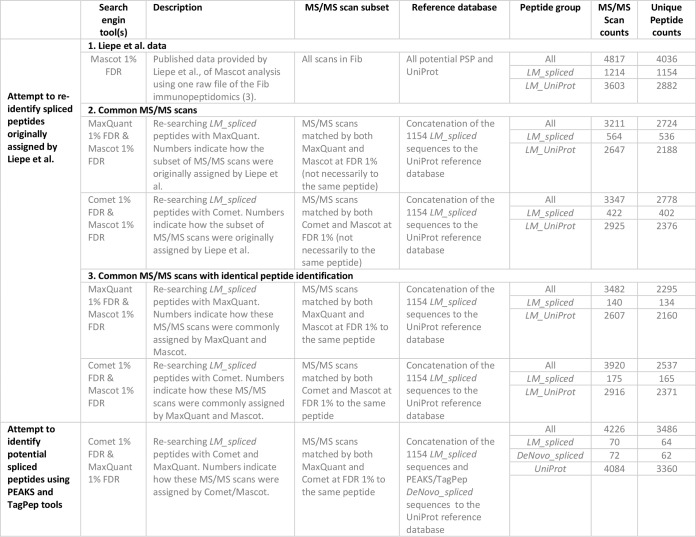
Summary of the level of agreement in scan matching and peptide identifications between Mascot (from Liepe et al.), MaxQuant, and Comet for the different subset of peptides identified in one raw file of immunopeptidomics data form the Fib dataset

To increase the coverage of possible spliced peptides, we searched three additional raw files of HLA-Ip derived from the same Fib cells. In total, MaxQuant identified 202 (17.5%) and Comet 235 (20.4%) peptides as *LM_spliced* sequences (supplemental Table 4). Compared with PSMs assigned as UniProt, peptides assigned as *LM_spliced* were characterized by lower Andromeda score for the best MS/MS spectrum (supplemental Fig. 3*A*), lower Andromeda score difference to the second best identified peptide (supplemental Fig. 3*B*), higher absolute precursor mass deviation (supplemental Fig. 3*C*), fewer peaks matching to the predicted fragmentation spectrum (supplemental Fig. 3*D*), lower fraction of total MS/MS peak intensity matched (supplemental Fig. 3*E*), and a larger fraction of singly charged MS/MS spectra matched (supplemental Fig. 3*F*). In agreement to our previous analysis employing a single Fib raw file, the combined analysis of four files showed similar results; the group of MS/MS scans matched to a spliced peptide from Liepe *et al.* showed a highly inflated disagreement (43.0%) compared with the UniProt scans (1.0%) (supplemental Fig. 3*G*). Altogether, these results indicate the matches to most *LM_spliced* peptides are of lower quality.

##### Spliced Peptides From Liepe et al. Conflict With Modified UniProt Peptides

Next, we tested how the inclusion of variable modifications in the searches influences the identification rates in the different groups. When adding variable modifications to the MaxQuant and Comet searches (deamidation of asparagine and glutamine, oxidation of methionine, acetylation of protein N-term), we observed that the percentage of spliced peptides decreased (supplemental Fig. 4*A*, supplemental Table 4). The MS/MS scans previously matched to spliced peptides were now matched to modified peptides in UniProt, mainly oxidations (71.4%) and deamidations (28.6%). Again, more conflicting sequences were found in the spliced peptide group (data not shown). If we restricted the analysis to the high quality PSMs, where both MaxQuant and Comet found the same peptide, the overall contribution of *LM_spliced* peptides shrinks further (supplemental Fig. 4*A*) to about 2% of the UniProt peptides.

##### Synthetic Peptide Searches

**Fig. 2.**
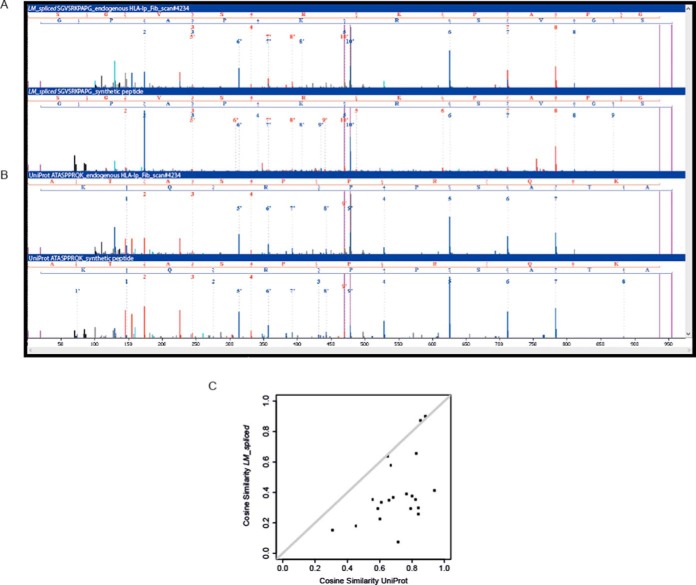
(*A*) Example of MS/MS annotation of endogenous HLA-Ip identified as an *LM_spliced* peptide (DHAQQPYSM), (*upper panel*) MS/MS of the synthetic counterpart of the *LM_spliced* (*lower panel*). (*B*) MS/MS annotation of the same endogenous HLA-Ip as an alternative UniProt peptide (DHRSEQSSM, *upper panel*), and MS/MS of the synthetic counterpart of the alternative UniProt peptide (*lower panel*). (*C*) The cosine similarity score calculated for the 21 pairs of MS/MS spectra of *LM_spliced* peptides and their synthetic counterparts and the pairs of the alternative sequences from UniProt and their synthetic peptides.

We selected 21 MS/MS scans from the Fib sample, where Liepe *et al.* matched a spliced peptide and MaxQuant matched a UniProt peptide. This selection was not biased to favor MaxQuant results, but we chose spectra that appeared to be typical for the group of spectra that produced conflicting results. For each of the 21 scans, we synthesized two peptides: one according to the *LM-spliced* identification and one for the UniProt alternative. We compared the MS/MS spectra of the synthetic peptides to the spectra of the endogenous peptides. As an example, we show the endogenous and synthetic spectra of the *LM_spliced* peptide SGVSRKPAPG (Fig. 2*A*) and its UniProt competitor peptide ATASPPRQK (Fig. 2*B*). All the 21 comparisons can be found in supplemental Fig. 5. Some *LM_spliced* peptides (for example, LENKKGKAL, RVTGALQKK) differ in only two positions from the alternative UniProt peptides (EINKKGKAL, RLSGALQKK), reflecting how similar spliced and UniProt matches can be. We computed the cosine-similarity score between synthetic and endogenous (*i.e.* original eluted HLA-Ip) spectra. For almost all the 21 cases, synthetic spectra from UniProt peptides fit better to the original MS/MS spectra compared with their *LM_spliced* peptides counterparts (Fig. 2*C*).

##### Alternative Pipeline to Estimate the Contribution of Spliced Peptides to the HLA-peptidome

**Fig. 3.**
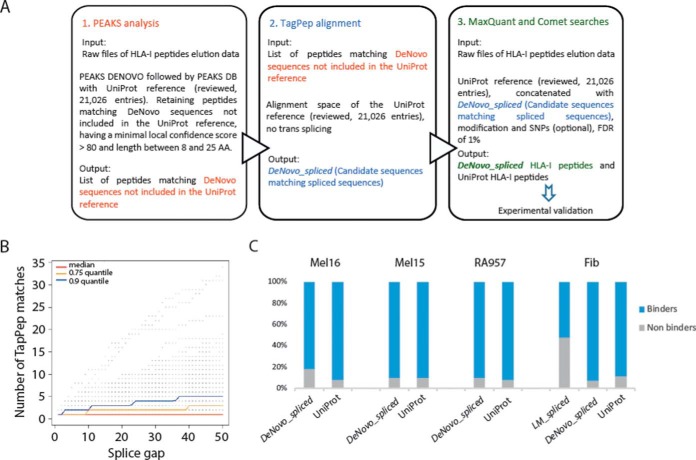
(*A*) Scheme of the *de novo* based pipeline to identify possible spliced peptides. (*B*) The splicing gap in relation to the number of TagPep matches. The number of TagPep matches is the number of returned hits with a splicing gap shorter than a given value. Hits with the same splicing position and splicing gap are merged and counted as one hit. (*C*) Distribution of 8–14-mer peptides predicted by NetMHCpan as binders and nonbinders among the *DeNovo_spliced* and UniProt peptides identified in Mel16, Mel15, RA957, and Fib samples, and in addition also *LM_spliced* peptides.

In order to shed more light on the detection of PSPs by MS, we implemented a different computational pipeline (Fig. 3*A*), which is based on *de novo* sequencing (25). This pipeline proceeds in three steps: 1) *de novo* sequencing of MS/MS spectra to retrieve only *de novo* sequences not found in UniProt; 2) flagging *de novo* sequences by the alignment tool TagPep as *DeNovo_spliced* peptides; and 3) adding the candidate *DeNovo_spliced* sequences to UniProt fasta protein/peptide reference database files and searching them with MaxQuant and Comet at FDR of 1% with variable modifications. The idea behind the last step is to match each MS/MS spectrum to either *DeNovo_spliced* or UniProt. Importantly, this computational pipeline bypasses the step of matching MS/MS spectra to a huge database of potential spliced peptides.

We used the PEAKS tool (13) for *de novo* sequencing of the immunopeptidomics MS/MS spectra from four different biological samples (see Experimental procedures section for more details). First, we estimated the error present in the PEAKS *de novo* sequences. We compared spectra matches assigned by both PEAKS and MaxQuant to UniProt peptides as a function of the PEAKS minimum local confidence score. For a minimum local confidence score higher than 80 the peptides assigned to the spectra by both, PEAKS and MaxQuant agreed in 80% of the cases (supplemental Fig. 6*A*). We can accept this level of performance since in our computational approach the proposed peptides are subsequently filtered by the consecutive MaxQuant or Comet analysis at FDR of 1%. Comparison for the UniProt sequences showed that PEAKS could identify about half the peptides as compared with MaxQuant at the given threshold of 80 (supplemental Fig. 6*B*). This could be expected due to deficient performance of the *de novo* sequencing given the often incomplete fragmentation of the HLAp peptides. Furthermore, we observed that PEAKS is not optimized for nontryptic peptides (see below).

Next we checked whether the identified *de novo* sequences that did not match a UniProt sequence could be PSPs by an in-house alignment tool called TagPep (14). TagPep is a very fast alignment tool employing efficient indexing. TagPep first matches the whole *de novo* peptide sequence to the protein database, and if there is no hit, it tries to match it with one splicing event (supplemental Data 5). The number of TagPep matches per *de novo* sequence depends on the allowed splice gap between the spliced protein fragments. Having no restrictions on the splice gap, 20% of random AA sequences of length 8–11 could be matched as a spliced peptide to the human proteome database. Fig. 3*B* shows that more than half of the *de novo* sequences produce a unique TagPep match with a splice gap of less than 20 AA, whereas 90% of the sequences have three matches or less. Only about 10% of the sequences have more than three matches and have many possible explanations.

Next, for each biological sample we created a separate fasta file by adding the list of the *DeNovo_spliced* candidate sequences to the UniProt fasta files (supplemental Data 5). All MS/MS spectra from each immunopeptidomics sample were subsequently matched against these fasta databases using the sequence search tools MaxQuant and Comet. To test our pipeline we used previously published immunopeptidomics high quality datasets (Mel15, Mel16, Fib, and RA957 samples), which represent a variety of HLA binding specificities (supplemental Table 1). For the Fib, RA957, Mel15, and Mel16 samples we identified 122 (175, 93), 165 (213, 110), 694 (839, 502), and 115 (163, 55) spliced peptides with MaxQuant (Comet, consensus of MaxQuant and Comet), respectively. The MaxQuant-Comet consensus *DeNovo_spliced* identifications constitutes 2.2%, 1.4%, 2.8%, and 0.7% of the identified immunopeptidome (supplemental Table 5). Only six (5) *LM_spliced* peptides were re-identified by our pipeline as *DeNovo_spliced* using MaxQuant (Comet) (supplemental Table 4). Most of the *DeNovo_spliced* peptides identified by MaxQuant-Comet were predicted to bind to the HLA-molecules (Fig. 3*C*). Moreover, we could see evidences of the expected binding motifs in the *DeNovo_spliced* peptides found by our pipeline (supplemental Fig. 7), even if the number of peptides is significantly smaller than for the predicted spliced peptides of Liepe *et al.* (3). The differences between the binding specificities of *DeNovo_spliced* and UniProt peptides, as seen in case of the HLA-B27:05 in Mel15, are most likely related to a bias against identification of HLA-B27 peptides in PEAKS (supplemental Fig. 8), which has difficulty identifying such nontryptic peptides.

Compared with the UniProt peptides, the *DeNovo_spliced* peptides found by our pipeline have a slightly higher absolute mass error and lower delta score, but they have very similar score and charge distribution. However, compared with the spliced peptides found by Liepe *et al.*, the *DeNovo_spliced* peptides have better match characteristics: lower absolute mass error, higher Andromeda scores and delta scores, higher number of matching ions, and fewer singly charged PSMs (supplemental Fig. 3*A*–3*F*).

##### Sequence Variants and Spliced Peptide Conflicts

Next, we searched for single AAs variations obtained by exome sequencing of the Mel15 and Mel16 samples (10). The spliced peptides found by TagPep for the Mel15 and Mel 16 samples were added to the fasta file used by Bassani-Sternberg *et al.*, which contained all *Ensemble* human protein sequences and the sequence variants identified by exome sequencing. Out of the 809 *DeNovo_spliced* peptides, 19 (2.3%) unique peptides had the same sequence as an endogenous HLA-Ip with a single AA variation (supplemental Data 17). These results highlight the needs to carefully evaluate spliced peptides identified by MS/MS and make sure that they do not have a different, potentially simpler explanation.

##### Characterization of the Splicing Events

**Fig. 4.**
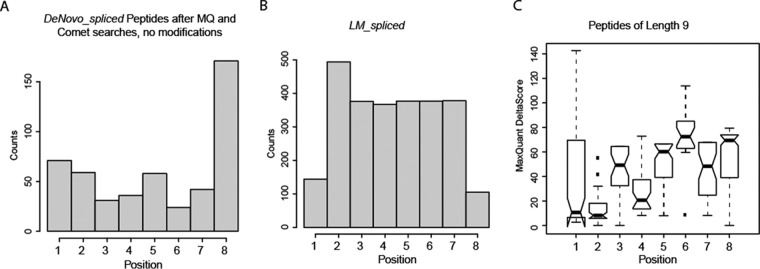
(*A*) Histogram of splicing positions within 9-mers for the *DeNovo_spliced* peptides identified by both Comet and MaxQuant. (*B*) Histogram of *LM_spliced* peptides splicing positions. (*C*) MaxQuant delta scores as a function of the splicing position within the 9-mers *DeNovo_spliced* peptides identified by both Comet and MaxQuant.

Lastly, we found that in many *DeNovo_spliced* peptides, identified by both MaxQuant and Comet, the splicing position is at the N- and C termini (Fig. 4*A*) in contrast to the *LM_spliced* peptides (Fig. 4*B*). It could also be possible that single AA abundant in the proteasome or during sample processing could be attached to the peptide termini. This effect is known as transpeptidation and was observed in tryptic samples (26). On the other hand, the distribution of MaxQuant delta scores as a function of splice position indicates that spliced peptide matches with N-terminal splice positions are on average more ambiguous than others (Fig. 4*C*).

## DISCUSSION

Examples of proteasomal-spliced peptides have been reported and some of them were shown to be immunogenic (4–9, 16, 27–29). A true estimation of the contribution of spliced peptides to the global immunopeptidome is critical in order to fundamentally understand the biological pathways involved. Consequently, advanced computational and experimental tools must be developed and benchmarked to facilitate their confident identification.

Liepe *et al.* (3) were the first to attempt to find PSPs by means of MS on a large scale. Their report concluded that about 30% of the HLA-Ip are produced by proteasomal splicing by transpeptidation of two noncontiguous fragments of a parental protein (cis-splicing). Our reanalysis of their results revealed that their approach led to the identification of PSPs candidates that did not fit the consensus binding motifs, while the nonspliced UniProt HLA-Ip identified in the same experiment did. Additional parameters related to their MS/MS spectrum matches suggest that many of the spliced peptide matches reported in Liepe *et al.* are ambiguous and were ruled out when we used different search engines and included common PTMs or sequence variants in the search. We postulate that because of the huge search space of potential spliced peptide, the bioinformatics approach applied by Liepe *et al.* led to uncontrollable propagation of false positives. The effect of database size and the increased likelihood of false positive identifications in proteogenomics applications have been thoroughly reviewed in (30), and these concepts are relevant here as well. Therefore, the true contribution of spliced peptides to the immunopeptidome has yet to be defined.

In a typical peptidomics setting, we match MS/MS spectra against a large set of theoretical peptide spectra, most of which are not present in the sample. This endeavor produces two types of PSMs: true matches and false positives. False positives are very common especially for spliced peptides since these peptides can produce similar MS/MS spectra to UniProt peptides with similar match scores. For example, the potential spliced peptide KRI-PLPTKK only differs from its UniProt counterpart RIKPLPTKK by a permutation of the first three AA. Because of absence of fragmentation in the region of the first three AAs in this example, their order cannot be determined. Furthermore, when using a very large proteasomal spliced peptide database there is an elevated chance that a potential spliced peptide will have a very similar spectrum to the UniProt peptide and produce a higher match score. Even if a spectrum has no match in the UniProt database (*e.g.* when it originates from a modified peptide, sequence variant, or contaminant not considered in the search), it may still match a spliced peptide with a score that is significant.

The error in the multiple testing of MS/MS searches is controlled by using decoy database in order to calculate the FDR (31, 32). One assumption behind this target-decoy approach is that the scores of the decoy peptides reflect the scores of wrongly assigned PSMs. When using decoys for spliced peptides, their similarity with the UniProt sequences may be lost, and one would have to carefully evaluate whether the assumption mentioned above still holds. If it does not hold, the target-decoy approach might underestimate the FDR and lead to many false positives especially for large spliced peptide databases.

Trans-splicing of fragments derived from two source proteins that happen to be present in the same proteasome complex at the same time, is unlikely to happen on a large scale, hence we focused our study on cis-splicing events. To overcome biases related to searching all possible cis-splice peptides, we developed an alternative workflow based on *de novo* sequencing and subsequent verification with multiple search tools including the most prevalent AA modifications and sequence variants detected by exome sequencing. We found that 1–3% of the high-quality PSMs originate from potential proteasome cis-spliced peptides. These peptides fitted the HLA consensus binding motifs and had good spectrum match properties. Given that our *de novo* sequencing approach finds about half of the peptides compared with a UniProt sequence search, we can say that the maximal amount of spliced peptide candidates is 2–6%. This doubling is just a very rough estimate and does not mean that the number of spliced peptides would double as well. Our approach focuses on the high quality spectra required for *de novo* sequencing. By including more low quality spectra, we would not only increase the number of spliced peptides, but also the ambiguity of the additional spliced peptide matches. However, MS/MS-based approaches cannot ultimately determine the creation mechanism of these peptides, and different sequence interpretations may also be possible. For example, a significant number of detected HLA-Ip originates from transcripts, which do not fall into a UniProt protein coding region (33), and these noncanonical peptides could be misinterpreted as PSPs. Other ambiguities may be due to post-translational or chemical peptide modifications not considered in the search. Therefore, we recommend to consider the most prominent chemical or posttranslational protein modifications in the MS/MS search. If these modifications are not known, open modification search tools (34, 35) could be applied. Overall, our results present an upper bound for the proportion of cis-spliced peptides, and the true contribution of such PSPs to the HLA-I ligandome may be much smaller. Extensive *in vitro* validation assays with purified proteasomes and using controlled cellular assays are required to assure that any of the proposed sequences are actually generated by splicing events *in vivo*.

## DATA AVAILABILITY

MS raw files of HLA-Ip isolated from two melanoma tissues, Mel15 (16 raw files) and Mel16 (12 raw files) (10), RA957 B cell line (four raw files) (11), and fibroblast (Fib) cells (four raw files) (2), included in datasets PXD004894, PXD005231 and PXD000394, respectively, were downloaded from the Proteome-Xchange Consortium via the PRIDE partner repository (12). The raw files and MaxQuant output tables related to the analysis of synthetic peptides used for the comparison of MS/MS annotations of endogenous HLA-Ip and their synthetic counterparts have been deposited to the Proteome-Xchange Consortium via the PRIDE partner repository with the dataset identifier PXD010793.

## Supplementary Material

supplemental Table 1

## Footnotes

^1^ The abbreviations used are:

HLA: human leukocyte antigen
HLA-Ip: HLA class I binding peptides
FDR: false discovery rate
FP: false positive
AA: amino acid
LC: liquid chromatography
MS: mass spectrometry
MS/MS: tandem mass spectrometry
PSPs: proteasome-spliced peptides
*DeNovo_spliced*: spliced peptides identified by de-novo
*LM_spliced*: spliced peptides identified by Liepe et al
*LM_UniProt*: UniProt peptides identified by Liepe et al
PSM: peptide spectrum match.
