# Supplementary material for: Estimating the Contribution of Proteasomal Spliced Peptides to the HLA-I Ligandome*
Source: Mol Cell Proteomics. 2018 Sep 5;17(12):2347–57. doi: 10.1074/mcp.RA118.000877 (PMC6283289; doi:10.1074/mcp.RA118.000877)
Supplement: supplemental Table 1 [file RA118.000877_index.html]

Supplement to Estimating the Contribution of Proteasomal Spliced Peptides to the HLA-I Ligandome | Molecular & Cellular Proteomics

## Supplemental Data

- Supplemental Figures 1-8 - Supplemental Figures 1-8
- Supplemental Tables 1-5 - Supplemental Tables 1-5
- Supplemental Legends - Supplemental Legends clean
- Supplemental Data 1 - Supplemental Data 1
- Supplemental Data 2 - Supplemental Data 2
- Supplemental Data 3 - Supplemental Data 3
- Supplemental Data 4 - Supplemental Data 4
- Supplemental Data 5 - Supplemental Data 5
- Supplemental Data 6 - Supplemental Data 6
- Supplemental Data 7 - Supplemental Data 7
- Supplemental Data 8 - Supplemental Data 8
- Supplemental Data 9 - Supplemental Data 9
- Supplemental Data 10 - Supplemental Data 10
- Supplemental Data 11 - Supplemental Data 11
- Supplemental Data 12 - Supplemental Data 12
- Supplemental Data 13 - Supplemental Data 13
- Supplemental Data 14 - Supplemental Data 14
- Supplemental Data 15 - Supplemental Data 15
- Supplemental Data 16 - Supplemental Data 16
- Supplemental Data 17 - Supplemental Data 17
