# Supplementary material for: Estimating the Contribution of Proteasomal Spliced Peptides to the HLA-I Ligandome*
Source: Mol Cell Proteomics. 2018 Sep 5;17(12):2347–57. doi: 10.1074/mcp.RA118.000877 (PMC6283289; doi:10.1074/mcp.RA118.000877)
Supplement: supplemental Table 1 [file 138326_2_supp_192533_ph4q2m.pdf]

Supplemental Table 1.

	**HLA-A**	**HLA-A**	**HLA-B**	**HLA-B**	**HLA-C**	**HLA-C**
**Fib**	A*03:01	A*23:01	B*08:01	B*15:18	C*07:02	C*07:04
**RA957**	A*02:20	A*68:01	B*35:03	B*39:01	C*04:01	C*07:02
**Mel15**	A*03:01	A*68:01	B*27:05	B*35:03	C*02:02	C*04:01
**Mel16**	A*01:01	A*24:02	B*07:02	B*08:01	C*07:01	C*07:02

Supplemenal Table 2

MaxQuant	Name	Description
	Raw file	The name of the RAW file the mass spectral data was read from.
	Scan number	The RAW-file derived scan number of the MS/MS spectrum.
	Scan index	The consecutive index of the MS/MS spectrum.
	Sequence	The identified AA sequence of the peptide.
	Length	The length of the sequence stored in the column "Sequence".
	Modifications	Post-translational modifications contained within the identified peptide sequence.
	Modified sequence	Sequence representation including the post-translational modifications (abbreviation of the modification in brackets before the modified AA). The sequence is always surrounded by underscore characters ('_').
	Deamidation (NQ) Probabilities	Sequence representation of the peptide including PTM positioning probabilities ([0..1], where 1 is best match) for 'Deamidation (NQ)'.
	Oxidation (M) Probabilities	Sequence representation of the peptide including PTM positioning probabilities ([0..1], where 1 is best match) for 'Oxidation (M)'.
	Acetyl (Protein N-term)	0=no modification; number indicate the number of modifications per peptide.
	Deamidation (NQ)	0=no modification; number indicate the number of modifications per peptide.
	Oxidation (M)	0=no modification; number indicate the number of modifications per peptide.
	Proteins	The identifiers of the proteins the identified peptide is associated with.
	Charge	The charge state of the precursor ion.
	Fragmentation	The type of fragmentation used to create the MS/MS spectrum. HCD – High energy Collision induced Dissociation.
	Mass analyzer	The mass analyzer used to record the MS/MS spectrum.FTMS – Fourier transform ICR or orbitrap cell.
	Type	The type of precursor ion as identified by MaxQuant. ISO –isotopic cluster.PEAK – single peak. MULTI – labeling cluster.
	Scan event number	Scan event number
	Isotope index	Isotope index
	m/z	The mass-over-charge of the precursor ion.
	Mass	The charge corrected mass of the precursor ion.
	Mass Error [ppm]	Mass error of the recalibrated mass-over-charge value of the precursor ion in comparison to the predicted monoisotopic mass of the identified peptide sequence.
	Simple Mass Error [ppm]	The precursor mass as it appears in the scan header and is an imprecise estimate
	Retention time	The uncalibrated retention time in minutes where the MS/MS spectrum has been acquired.
	PEP	Posterior Error Probability of the identification. This value essentially operates as a p-value, where smaller is more significant.

	Score	Andromeda score for the best associated MS/MS spectrum.
	Delta score	Score difference to the second best identified peptide with a different amino acid sequence.
	Matches	The species of the peaks in the fragmentation spectrum after TopN filtering.
	Intensities	The intensities of the peaks in the fragmentation spectrum after TopN filtering.
	Mass Deviations [Da]	The mass deviation of each peak in the fragmentation spectrum in absolute mass units.
	Mass Deviations [ppm]	The mass deviation of each peak in the fragmentation spectrum in parts per million.
	Masses	The masses-over-charge of the peaks in the fragmentation spectrum.
	Number of Matches	The number of peaks matching to the predicted fragmentation spectrum.
	Intensity coverage	The fraction of intensity in the MS/MS spectrum that is annotated.
	Peak coverage	The fraction of peaks in the MS/MS spectrum that are annotated.
	Neutral loss level	How many neutral losses were applied to each fragment in the Andromeda scoring.
	Reverse	When marked with '+', this particular peptide was found to be part of a protein derived from the reversed part of the decoy database. These should be removed for further data analysis.
	All scores	Scores of additional hits matching the spectrum at a decreasing order
	All sequences	sequences matching the spectrum at a decreasing order
	All modified sequences	sequences with modification marked matching the spectrum at a decreasing order
	id	A unique (consecutive) identifier for each row in the msms table, which is used to cross-link the information in this file with the information stored in the other files.
**Comet**	Spectrum	raw file name.scane number.charge
	ScanNumber	The RAW-file derived scan number of the MS/MS spectrum.
	Charge	The charge state of the precursor ion.
	PrecMz	The charge corrected mass of the precursor ion.
	MassDiff	Mass error of the mass-over-charge value of the precursor ion in comparison to the predicted monoisotopic mass of the identified peptide sequence.
	PeptideAnnot	Sequence representation including the post-translational modifications
	PeptideSeq	The identified AA sequence of the peptide.
	Protein	The identifiers of the proteins the identified peptide is associated with.
	deltacnstar	Comet deltacnstar score for best hit (PMID: 24226387)
	spscore	Comet spscore score for best hit (PMID: 24226387)
	xcorr	Comet xcorr score for best hit (PMID: 24226387)
	deltacn	Comet deltacn score for best hit (PMID: 24226387)
	expect	Comet expectaion value score for best hit (PMID: 26115965)
	sprank	Comet sprank score for best hit (PMID: 24226387)

	Rank	Comet PSM rank (best hit = rank 1)
**PEAKS**	**Scan**	The scan number. The scan number is a unique index for tandem mass spectra in the data.
	**Source.File**	The name of the raw file processed.
	**Peptide**	The amino acid sequence of the peptide, as determined in PEAKS Search. A modified residue is followed by a pair of parentheses enclosing the modification mass.
	**Tag.Length**	The length of the longest sequence tag in the peptide. When low confidence residues are reduced to mass tags, the remaining residues become sequence tags. The length of the longest tag is reported in this column. Refer to Mass Tags.
	**ALC....**	The significance score of the peptide identified by database searching or by de novo sequencing for de novo only peptides. The ALC score is the average of local confidence score of all of the residues in a de novo sequence.
	**length**	The number of amino acids in the peptide sequence.
	**m.z**	The mass-over-charge of the precursor ion.
	**z**	The charge state of the precursor ion.
	**RT**	The retention time (elution time) for the spectrum as recorded in the data.
	**Area**	The area under the curve of the peptide feature found at the same m/z and retention time as the MS/MS scan. This can be used as an indicator of the abundance of the peptide.
	**Mass**	The monoisotopic mass of the peptide.
	**ppm**	The precursor mass error, calculated as 10^6 × (precursor mass - peptide mass) / peptide mass.
	**Accession**	The proteins supported by the peptide, separated by ':' if the peptide supports multiple proteins. If none of the proteins that the peptide supports pass the protein filters, the accession field will be empty.
	**PTM**	The types and the numbers of the modifications present in the peptide shown as color-coded icons.
	**local.confidence....**	Local confidence score for each amino acid in a de novo sequence. The local confidence score ranges from 0% to 99%, indicating how confident the algorithm considers a particular amino acid as correctly sequenced.
	**tag....0..**	The best matching de novo sequencing tag.
	**mode**	The fragmentation mode in which de novo sequencing is performed by the algorithm.
	**tagpep.hits**	Accession number and possition of splicing.

Supplemental Table 3.

mgfFile	*LM-spliced*	*LM-spliced* Similarity score	UniProt	UniProt Similarity score
3448.mgf	KWKVTASVV	0.07	RTGKVTVEK	0.72
3676.mgf	KKLMPGKKEL	0.87	ELRQKKKEL	0.86
3818.mgf	KGGCKPKAAP	0.22	GTVKPNANR	0.60
4045.mgf	KRQPESGIK	0.29	RQKPESLGK	0.59
4234.mgf	SGVSRKPAPG	0.37	ATASPPRQK	0.69
4277.mgf	IVDKMAPGSASK	0.18	IVDDRGRSTGK	0.45
4280.mgf	LENKKGKAL	0.63	EINKKGKAL	0.65
4289.mgf	KAEKSHDEPG	0.35	ATEDSQRYK	0.66
4301.mgf	TRSPVKQPR	0.90	RTSPVPRQK	0.89
4430.mgf	LGSEHRLSNK	0.30	AVSEHRLSNK	0.84
4566.mgf	KTQTMKTPG	0.41	ATKTPMSQK	0.94
4620.mgf	KRITKEINEK	0.65	RLKTKENLEK	0.83
4650.mgf	KQGQPVLKK	0.58	KANPQVLKK	0.67
4668.mgf	KFLGMSFNKK	0.25	RIQEHFNKK	0.84
4696.mgf	KQQIHVKKK	0.34	RLIDHVKKK	0.61
4716.mgf	KISNACPQTK	0.35	VVM(+15.995)RDPQTK	0.82
4776.mgf	KQENPPAVQQK	0.39	KTTDRYVQQK	0.77
4806.mgf	KKQKQQSPG	0.38	ATSQQPLRK	0.80
4897.mgf	HGPLTATGTK	0.15	RVYGTTGTK	0.31
4969.mgf	VLSTESPASK	0.29	VVM(+15.995)RDPASK	0.79
4996.mgf	RVTGALQKK	0.35	RLSGALQKK	0.56
	mean	0.40		0.70

	LM-spliced	LM-spliced Similarity score
4031.mgf	LENKKGKSL	0.66
4783.mgf	KRIPLPTKK	0.92
4819.mgf	ATSQQKRLP	0.77
	mean	0.69

Supplemental Table 4:

	PeptidesMatchedBy	ScansMatchedBy	PSMSubset	VarModif	ScanCounts	PeptideCounts
1	MaxQuant	MaxQuant	all		16651	6956
2	MaxQuant	MaxQuant	LM_spliced		423	202
3	MaxQuant	MaxQuant	DeNovo_spliced		331	140
4	MaxQuant	MaxQuant	LM_spliced&DeNovo_spliced		16	6
5	MaxQuant	MaxQuant	UniProt		15897	6614
6	Comet	Comet	all		21598	8307
7	Comet	Comet	LM_spliced		568	235
8	Comet	Comet	DeNovo_spliced		448	180
9	Comet	Comet	LM_spliced&DeNovo_spliced		14	5
10	Comet	Comet	UniProt		20582	7892
11	Comet&MaxQuant	Comet&MaxQuant	all		11638	5027
12	Comet&MaxQuant	Comet&MaxQuant	LM_spliced		174	93
13	Comet&MaxQuant	Comet&MaxQuant	DeNovo_spliced		229	104
14	Comet&MaxQuant	Comet&MaxQuant	LM_spliced&DeNovo_spliced		11	5
15	Comet&MaxQuant	Comet&MaxQuant	UniProt		11235	4830
16	MaxQuant	MaxQuant	all	Ac,De,Ox	14748	6055
17	MaxQuant	MaxQuant	LM_spliced	Ac,De,Ox	339	160
18	MaxQuant	MaxQuant	DeNovo_spliced	Ac,De,Ox	276	122
19	MaxQuant	MaxQuant	LM_spliced&DeNovo_spliced	Ac,De,Ox	14	6
20	MaxQuant	MaxQuant	UniProt	Ac,De,Ox	14133	5773
21	Comet	Comet	all	Ac,De,Ox	21822	7952
22	Comet	Comet	LM_spliced	Ac,De,Ox	535	203
23	Comet	Comet	DeNovo_spliced	Ac,De,Ox	435	175
24	Comet	Comet	LM_spliced&DeNovo_spliced	Ac,De,Ox	15	5
25	Comet	Comet	UniProt	Ac,De,Ox	20852	7574
26	Comet&MaxQuant	Comet&MaxQuant	all	Ac,De,Ox	10226	4339
27	Comet&MaxQuant	Comet&MaxQuant	LM_spliced	Ac,De,Ox	126	68
28	Comet&MaxQuant	Comet&MaxQuant	DeNovo_spliced	Ac,De,Ox	188	93
29	Comet&MaxQuant	Comet&MaxQuant	LM_spliced&DeNovo_spliced	Ac,De,Ox	9	5
30	Comet&MaxQuant	Comet&MaxQuant	UniProt	Ac,De,Ox	9912	4178

Supplemental Table 5:

	Sample	PeptidesMatchedBy	ScansMatchedBy	PSMSubset	VarModif	ScanCounts	PeptideCounts
1	Fib	Comet	Comet	all	Ac,De,Ox	21287	7749
2	Fib	Comet	Comet	DeNovo_spliced	Ac,De,Ox	435	175
3	Fib	Comet	Comet	UniProt	Ac,De,Ox	20852	7574
4	Fib	MaxQuant	MaxQuant	all	Ac,De,Ox	14409	5895
5	Fib	MaxQuant	MaxQuant	DeNovo_spliced	Ac,De,Ox	276	122
6	Fib	MaxQuant	MaxQuant	UniProt	Ac,De,Ox	14133	5773
7	Fib	Comet,MaxQuant	Comet,MaxQuant	all	Ac,De,Ox	10100	4271
8	Fib	Comet,MaxQuant	Comet,MaxQuant	DeNovo_spliced	Ac,De,Ox	188	93
9	Fib	Comet,MaxQuant	Comet,MaxQuant	UniProt	Ac,De,Ox	9912	4178
10	Mel15	Comet	Comet	all	Ac,De,Ox	252338	28999
11	Mel15	Comet	Comet	DeNovo_spliced	Ac,De,Ox	7023	839
12	Mel15	Comet	Comet	UniProt	Ac,De,Ox	245315	28160
13	Mel15	MaxQuant	MaxQuant	all	Ac,De,Ox	191355	23239
14	Mel15	MaxQuant	MaxQuant	DeNovo_spliced	Ac,De,Ox	5304	694
15	Mel15	MaxQuant	MaxQuant	UniProt	Ac,De,Ox	186051	22545
16	Mel15	Comet,MaxQuant	Comet,MaxQuant	all	Ac,De,Ox	138848	18164
17	Mel15	Comet,MaxQuant	Comet,MaxQuant	DeNovo_spliced	Ac,De,Ox	3273	502
18	Mel15	Comet,MaxQuant	Comet,MaxQuant	UniProt	Ac,De,Ox	135575	17662
19	Mel16	Comet	Comet	all	Ac,De,Ox	98526	13924
20	Mel16	Comet	Comet	DeNovo_spliced	Ac,De,Ox	860	163
21	Mel16	Comet	Comet	UniProt	Ac,De,Ox	97666	13761
22	Mel16	MaxQuant	MaxQuant	all	Ac,De,Ox	76520	11450
23	Mel16	MaxQuant	MaxQuant	DeNovo_spliced	Ac,De,Ox	645	115
24	Mel16	MaxQuant	MaxQuant	UniProt	Ac,De,Ox	75875	11335
25	Mel16	Comet,MaxQuant	Comet,MaxQuant	all	Ac,De,Ox	48557	7688
26	Mel16	Comet,MaxQuant	Comet,MaxQuant	DeNovo_spliced	Ac,De,Ox	278	55
27	Mel16	Comet,MaxQuant	Comet,MaxQuant	UniProt	Ac,De,Ox	48279	7633
28	RA957	Comet	Comet	all	Ac,De,Ox	39545	14313
29	RA957	Comet	Comet	DeNovo_spliced	Ac,De,Ox	565	213
30	RA957	Comet	Comet	UniProt	Ac,De,Ox	38980	14100
31	RA957	MaxQuant	MaxQuant	all	Ac,De,Ox	25660	10831
32	RA957	MaxQuant	MaxQuant	DeNovo_spliced	Ac,De,Ox	420	165
33	RA957	MaxQuant	MaxQuant	UniProt	Ac,De,Ox	25240	10666
34	RA957	Comet,MaxQuant	Comet,MaxQuant	all	Ac,De,Ox	17705	7801
35	RA957	Comet,MaxQuant	Comet,MaxQuant	DeNovo_spliced	Ac,De,Ox	247	110
36	RA957	Comet,MaxQuant	Comet,MaxQuant	UniProt	Ac,De,Ox	17458	7691
