# Supplementary material for: Estimating the Contribution of Proteasomal Spliced Peptides to the HLA-I Ligandome*
Source: Mol Cell Proteomics. 2018 Sep 5;17(12):2347–57. doi: 10.1074/mcp.RA118.000877 (PMC6283289; doi:10.1074/mcp.RA118.000877)
Supplement: supplemental Table 1 [file 138326_2_supp_192516_p24q22.pdf]

Supplemental Table 1: HLA typing information.

Supplemental Table 2: Explanation of the column headers in MaxQuant, Comet and PEAKS result files.

Supplemental Table 3: List of 21 MS/MS scans annotated as LM_spliced as UniProt and of the three *LM_spliced* peptides, including the similarity scores to their synthetic counterparts.

Supplemental Table 4: Number of identified MS/MS scans and unique peptides for the four Fib samples applying different search tools and modification settings.

Supplemental Table 5: Number of identified MS/MS scans and unique peptides for the Mel15, Mel16 and RA957 samples applying different search tools and modification settings.

Supplemental Data 1: PEAKS “all de novo candidate” output table for the Fib dataset.
Supplemental Data 2: PEAKS “all de novo candidate” output table for the Mel15 dataset.
Supplemental Data 3: PEAKS “all de novo candidate” output table for the Mel16 dataset.
Supplemental Data 4: PEAKS “all de novo candidate” output table for the RA957 dataset.

Supplemental Data 5: Filtered list of PEAKS identification, including matches to UniProt, de-novo PSMs and the TagPep matches for the Fib, Mel16, Mel15 and RA957 data.

Supplemental Data 6: A) List of identified spliced (psp) and non-spliced peptides (pcp) in the Fib sample (20130504_EXQ3_MiBa_SA_Fib-2.dat), including the sequences, mapping of the MS/MS scans and their Mascot ion scores as provided by Liepe et al. B) same list as in A including PSMs from the reanalysis with MaxQuant. C) List of identified spliced and D) non-spliced peptides from the GR-LCL 2D dataset form Liepe et al.

Supplemental Data 7: MQ_Fib_Liepe_UP_msms.txt :
MaxQuant msms.txt file for the 4 Fib samples with variable modifications M-oxid and Nterm-acetyl. database UniProt, LM_spliced, DeNovo_spliced, DeNovo_nonspliced

Supplemental Data 8: MQ_Fib_Liepe_UP_modif_msms.txt:
MaxQuant msms.txt file for the 4 Fib raw files with variable modifications M_oxid, Nterm-acetyl, QN_deamid: database UniProt, LM_spliced, DeNovo_spliced, DeNovo_nonspliced

Supplemental Data 9: MQ_Mel15_modif_msms.txt
MaxQuant msms.txt file for the 16 Mel15 raw files with variable modifications M_oxid, Nterm-acetyl, QN_deamid: database UniProt, LM_spliced, DeNovo_spliced, DeNovo_nonspliced

Supplemental Data 10: MQ_Mel16_modif_msms.txt
MaxQuant msms.txt file for the 12 Mel16 raw files with variable modifications M_oxid, Nterm-acetyl, QN_deamid: database UniProt, LM_spliced, DeNovo_spliced, DeNovo_nonspliced

Supplemental Data 11: MQ_RA957_modif_msms.txt
MaxQuant msms.txt file for the 4 RA957 raw files with variable modifications M_oxid, Nterm-acetyl, QN_deamid: database UniProt, LM_spliced, DeNovo_spliced, DeNovo_nonspliced

Supplemental Data 12: 20130504_EXQ3_MiBa_SA_Fib_all.csv:
Comet PSM text file for the 4 Fib raw files with variable modifications M_oxid, Nterm-acetyl. database UniProt, LM_spliced, DeNovo_spliced, DeNovo_nonspliced

Supplemental Data 13: 20130504_EXQ3_MiBa_SA_Fib_all_modif.csv:
Comet PSM text file for the 4 Fib raw files with variable modifications M_oxid, Nterm-acetyl, QN_deamid, KRDE_methyl. database UniProt, LM_spliced, DeNovo_spliced, DeNovo_nonspliced

Supplemental Data 14: Co_Mel15_modif_msms.csv
Comet PSM text file for the 16 Mel15 raw files with variable modifications M_oxid, Nterm-acetyl, QN_deamid, KRDE_methyl. database UniProt, LM_spliced, DeNovo_spliced, DeNovo_nonspliced

Supplemental Data 15: Co_Mel16_modif_msms.csv

Comet PSM text file for the 12 Mel16 raw files with variable modifications M_oxid, Nterm-acetyl, QN_deamid, KRDE_methyl. database UniProt, LM_spliced, DeNovo_spliced, DeNovo_nonspliced

Supplemental Data 16: Co_RA957_modif_msms.csv

Comet PSM text file for the 4 RA957 raw files with variable modifications M_oxid, Nterm-acetyl, QN_deamid, KRDE_methyl. database UniProt, LM_spliced, DeNovo_spliced, DeNovo_nonspliced

Supplemental Data 17: List of peptides identified in Mel15 and Mel16 with SNP alterations when searched against personalized references including the TagPep hits.
